# Awareness and perceptions of women regarding human rights related to maternal health in rural Bangladesh

**DOI:** 10.7189/jogh.09.010415

**Published:** 2019-06

**Authors:** Janet E Perkins, Ahmed Ehsanur Rahman, Abu Bakkar Siddique, Tapas Mazumder, Mohammad Rifat Haider, Shams El Arifeen

**Affiliations:** 1 University of Edinburgh, Edinburgh, UK; 2 Maternal and Child Health Division, International Centre for Diarrhoeal Disease Research (icddr,b), Dhaka, Bangladesh; 3 University of South Carolina, South Carolina, USA; * Joint first authors with equal contributions

## Abstract

**Background:**

The global development community has increasingly come to frame preventable maternal mortality and morbidity principally as a violation of women’s basic human rights, necessitating a human rights-based approach to be appropriately addressed. In this article, we explore how human rights are understood and perceived in relation to maternal health at the local level in rural Bangladesh. This is essential given the momentum at the global level to promote rights and apply rights-based approaches to maternal health.

**Methods:**

A community-based, cross-sectional household survey was conducted in three upazilas (sub-districts) of Brahmanbaria district, Bangladesh in 2018. A total of 1367 women with a birth outcome in the past 12 months were interviewed. Descriptive statistics were used to report the awareness and perceptions of human rights related to maternal health. Multiple logistic regression was used to identify the associations between awareness and perceptions of human rights and background characteristics and, finally, with the use of skilled maternal health services.

**Results:**

Over two-thirds of women reported that they were aware that women have human rights related to maternal health. However, less than 10% were able to mention at least three specific human rights related to maternal health. Half of the women mentioned husbands as duty-bearers, while only 20% mentioned the government as a duty-bearer. One-third of women reported that they are able to realize their rights related to maternal health satisfactorily. Awareness and perceptions of human rights related to maternal health were significantly associated with higher educational attainment and wealth status. They were also associated with increased use of antenatal care.

**Conclusions:**

These findings suggest that interventions promoting the awareness of human rights related to maternal health would be appropriate within the communities of rural Bangladesh as part of a broader human rights-based approach to improving maternal health.

Despite the fact that nearly all deaths resulting from complications related to pregnancy and childbirth are entirely preventable, maternal mortality remains the second leading cause of death among women and girls of reproductive age worldwide [1,2]. Recognizing this, along with the global failure to adequately tackle the issue, the global development community has increasingly come to frame preventable maternal mortality and morbidity principally as a violation of women’s basic human rights. These include the right to life, be equal in dignity, education, be free to seek, receive and impart information, enjoy the benefits of scientific progress, be free from discrimination, and t enjoy the highest attainable standard of physical and mental health, including sexual and reproductive health [3]. States are legally obliged by international laws and commitments to respect, protect and fulfil these basic human rights [4].Ensuring that women are able to enjoy these rights is not only critical to their health and well-being, but also to that of newborns [5].

While the roots of this human rights reframing can be traced back to the International Conference on Population and Development (ICPD) in 1994, this narrative has accelerated in recent years [6]. This is reflected in key global guiding documents, including the historic resolution of the High Commissioner for Human Rights, denouncing preventable maternal mortality and morbidity as a violation of women’s human rights in 2009, followed by the issuance of technical guidance supporting countries to apply a human rights-based approach to improve maternal health in 2012 [3,7]. More recently, this paradigm has been further anchored in the Sustainable Development Goals (SDGs) and the Global Strategy for Women’s, Children’s and Adolescents’ Health, for which a high-level working group was tasked with securing political support for the human rights-related measures encapsulated therein [5,8].

Within this framing, a human rights-based approach to policy, strategy, and programming is required for improving maternal health. According to the UN common understanding, such an approach is characterized by the following key elements: 1) the goal of actions is the advancement of human rights as laid out in human rights instruments, 2) human rights principles (eg, universality, inalienability, participation, equity and non-discrimination and accountability) guide processes of implementation and 3) initiatives are taken to build the capacities of rights-holders to claim their rights and of duty-bearers to fulfil their obligations to respect, protect and fulfil human rights [9]. The High Level Working Group for the Health and Human Rights of Women, Children and Adolescents, co-hosted by the World Health Organization (WHO) and the Office of the High Commissioner for Human Rights (OHCHR), has recommended a framework of actions to be taken by states and other stakeholders in applying a human rights-based approach to improving the health and well-being of women, children and adolescents [8]. In addition to creating an enabling environment of policies, strategies and programmes, as well as ensuring the availability, accessibility, acceptability and quality (AAAQ) of health services, the framework of actions recommends partnering with people to enable them to realize their rights [8,10]. It calls for action at the local level to build the capacities of women as rights-holders to claim their rights, and of local actors as duty-bearers to support women to realize their rights. Raising awareness of women, families and communities is acknowledged as one of the fundamental steps towards developing capacities and creating an enabling platform. Indeed, in 2015, the World Health Organization (WHO) issued a recommendation for promotion of the awareness of human rights at the community level as a key intervention to improve maternal and newborn health, primarily as a matter of principle, despite the inconclusiveness of existing evidence to this effect [11].

Similar to many other low- and middle-income countries, Bangladesh has made considerable improvements in maternal health over the past two decades. However, the country continues to experience some of the highest maternal mortality rates in the world [12,13]. This can be traced, at least in part, to the critically low coverage of life-saving maternal health services. Indeed, only 37% pregnant women attend at least four antenatal care (ANC) contacts, half of the births are not attended by a skilled attendant and just 48% of women receive postnatal care from a skilled health care professional within the first two days after birth [13]. As per the access to health services framework presented by Peters e.al. (2009), a number of factors prevent women in Bangladesh and in other low-resource settings from accessing and availing health services related to safe pregnancy and childbirth. Many of these factors, at their root, reflect violations of women’s basic human rights, including the right to access sexual and reproductive health services, rights related to bodily autonomy and decision-making, the right to respectful maternity care, and the right to education and information [8].

Reflecting global trends, the maternal health strategy of Bangladesh has integrated a human rights-based approach, with the primary objective to ensure that all women are able to realize their rights during pregnancy, childbirth and the postpartum period [14]. However, despite these trends at the international and national level, the current evidence fails to shed light on the perceptions and understanding of human rights related to maternal heath, and how they are related to utilization of maternal health services at the local level. It is also important to understand the feasibility, acceptability and appropriateness of the promotion of such human rights in low-resource settings such as Bangladesh. In this article, we aim to understand women’s awareness of human rights related to maternal health, perceptions of the degree of women’s realization of these rights, and how this awareness and these perceptions are associated with the use of skilled maternal health services in rural Bangladesh. This will contribute to a better understanding of awareness and perceptions of rights in order to develop effective strategies to promote human rights and apply human rights-based approaches to maternal health.

## METHODS

### Study design and settings

We conducted a community-based, cross-sectional household survey in three upazilas (sub-districts) (Bijoynagar, Kasbah and Sarail) of Brahmanbaria district, Bangladesh in 2018. Brahmanbaria is located in the east-central region of Bangladesh and has nine upazilas, with a population of approximately 2.5 million. Each upazilas included in the study has an approximate population of 300 000. The economy of Brahmanbaria is based primarily on agriculture. No large-scale maternal and newborn health programmes were in place at the time of the survey. Table S1 in **Online Supplementary Document** outlines the population and health systems of the selected upazilas.

### Study population, sample size and sampling

Women with a recent history of birth (within the 12-month period preceding the survey) who were permanent residents of the selected sub-districts were eligible to be included in the study. We adopted stratified cluster sampling to identify eligible participants for the household survey. The selected sub-districts were considered as the strata, and villages (approximately 1000 populations) were regarded as clusters. We adopted probability proportional to size (PPS) sampling to select 20 villages (PPS clusters) from each of the selected sub-districts. All eligible respondents from the selected villages were approached for an interview using an interviewer administered structured questionnaire. A total of 1367 women with a recent history of birth were finally interviewed, with a non-response rate of approximately 1%.

### Data collection

The household survey was conducted between March and May of 2018. Initially, a sketch map was drawn for each of the selected villages representing boundaries, landmarks and household locations. All households within this sketch map were enumerated and listed. All women with a recent birth history were screened and identified from the listed households. In the second stage, an interviewer-administered structured questionnaire was used for interviewing all eligible women. The majority of the questions were adapted from the questionnaires used in Bangladesh Demographic and Health Survey (BDHS), Bangladesh Maternal Mortality Survey (BMMS), Multiple Indicator Cluster Survey (MICS) and other relevant studies [15-17]. Participants were first interviewed regarding their personal and socioeconomic information, including age, educational attainment, marital status, employment status etc. They were then asked regarding their awareness and practices related to maternal and newborn health, including awareness of and perceptions around the realization of human rights related to maternal health, and utilization of maternal health services.

For quality assurance, all data collection instruments were pre-tested in non-selected villages of the sub-districts under survey. Interviewers were recruited locally so that they would be familiar with the local context, culture and dialects. All interviewers received three days of training on the data collection tools, followed by a further three days of field practice prior to the commencement of data collection. The training was conducted by study investigators and master trainers with expertise in conducting household surveys. Refresher trainings were organized fortnightly during the survey. Field supervisors and managers ensured quality of data through spot checking, back checking filled questionnaires and providing feedback to the data collectors through review meetings.

### Data analysis

Data were analysed using Stata 13.0 (StataCorp LP, College Station, TX, USA). For sociodemographic characteristics, age and educational attainment of women were transformed into categorical variables. Due to small numbers, all other religions except ‘Muslim’ were grouped into one category and coded as ‘other’. We used the standard steps of principal component analysis to generate the socio-economic indices of the households that were interviewed, based on which the wealth quintile was generated [18,19]. Household-level variables such as household possessions; materials used for the construction of floor, wall, and roof; drinking water source; toilet facilities; and ownership of land and domestic animals were used to generate this index.

We used descriptive statistics (proportions) to report the indicators selected for reflecting awareness and perceptions regarding human rights related to maternal health as follows:

- **Awareness of humanrights related to maternal health:** Awareness in general that women have rights related to maternal health (during pregnancy, childbirth and after birth); awareness of the following specific human rights related to maternal health: the right to access maternal health services, the right to respectful maternity care, the right to information, the right to decide to seek health services autonomously, the right to family planning, and the right to be free from violence.
- **Awareness of duty-bearers with roles in ensuring the realization of rights related to maternal health:** Awareness of the following duty-bearers with obligations and responsibilities to respect, protect and fulfil human rights related to maternal health: government (identified as the principle duty-bearer in a human rights-based approach) and husbands, families and communities (identified as the moral duty-bearers in a human rights-based approach).
- **Perceptions around realization of human rights related to maternal health:** Perception that the woman herself is able to realize her rights related to maternal health; perception that women in the broader community are able to realize their rights related to maternal health.
- **Perceptions related to the roles played by duty-bearers:** Perception that duty-bearers (government, husbands, families and communities) satisfactorily meet their obligations to respect, protect and fulfil these human rights.

The estimates were stratified by the following background characteristics: age, education, religion, parity, involvement in income generating activities and wealth quintile. We then analysed the associations between awareness of human rights related to maternal health and perceptions regarding the realization of human rights and the roles of duty-bearers and background characteristics through binary logistic regression (presented with the unadjusted odd ratio (OR)). Effects of the covariates and confounders (background characteristics) were then adjusted through multiple logistic regression models and presented with adjusted odd ratios (AOR).

Finally, we analysed the associations between select awareness and perceptions indicators regarding human rights related to maternal health and utilization of maternal health services during the most recent pregnancy. For these analyses, awareness of human rights in general related to maternal health, awareness of at least three human rights related to maternal health, awareness of the role of government and husbands as duty-bearers, and perceptions regarding the personal realization of rights were considered explanatory variables. Women attending at least four ANC contacts from formal health care providers (HCP) and birth in the presence of a skilled birth attendant (SBA) were considered as outcome variables. Separate multiple logistic regression models were used to control for the effect of the covariates and confounders (background characteristics) and relationships were presented with AORs.

All OR and AORs are reported with 95% confidence intervals (CI). An association was considered significant if both the lower and upper limit of the CI were more or less than 1.

### Ethical approval and consent to participate

Ethical approval to conduct the study was obtained from the Institutional Review Board of icddr,b (PR-17088). Administrative approval was obtained from the central and local health authorities and managers prior to data collection.

Participation in the study was voluntary and no compensation was provided for participation. The consent form was translated into Bangla, which is the local language. All potential participants were fully informed regarding the objectives of the study and use of the data prior to the commencement of interviews. Written informed consent was obtained from each study participant prior to the interview. For participants with limited literacy, verbal informed consent was taken along with their thumb impression on the consent form. Verbal consent was audio-recorded. The participants were informed of their right to withdraw their participation at any time during the study, without showing any cause. They were also informed that refusing to participate in the study would not involve any penalty and would not influence the health services that she or her family receives. Privacy, anonymity and confidentiality of participants were strictly maintained, and all information was kept under lock and key.

## RESULTS

**Table 1** presents the background characteristics of the women who participated in the study. The mean age of women was 25 years (standard deviation = 5.2), with around two-thirds of the respondents between the age of 20-30. The participants had completed an average of 6.7 years of schooling. Nearly one-fifth of the respondents had completed less than five years of formal education, with more than half having completed 5-9 years, and another one-fifth having completed 10 or more years. The majority of respondents were Muslim (98%). Around one-third were the first-time mothers and one-fifth had four or more children. A negligible proportion of the women reported that they had been engaged in any income generating activity in the past 12 months.

**Table 1:** Background characteristics of the women with a recent history of birth (N = 1367)

Background characteristic	Percent
**Age (years):**	
15-19	9.7
20-24	38.8
25-30	27.9
31-35	15.7
35+	7.9
Mean age in years (SD)	25.3 (±5.2)
**Education:**	
Primary incomplete (0-4 years)	20.7
Primary complete to secondary incomplete (5-9 years)	58.4
Secondary complete or higher (10+ years)	20.9
Mean years of schooling (SD)	6.7 (3.4)
**Religion:**	
Muslim	97.4
Other (Hindu/Christian etc.)	2.6
**Number of living children:**	
1	30.1
2	27.6
3	19.3
4 or more	22.8
**Income generating activity:**	
Involved in the past 12 months	4.2
**Wealth quintile:**	
Lowest	20.0
Second	20.0
Middle	20.0
Fourth	20.0
Highest	20.0

SD – standard deviation

**Table 2** presents the levels of awareness and perceptions of women regarding human rights related to maternal health. More than two-thirds of women reported that they were aware that women have specific rights related to pregnancy, childbirth and afterbirth. However, only two-fifths of the women mentioned that women have the specific right to access quality maternal health services. Only one-fifth mentioned that they have the right to respectful maternity care from the health service providers. Less than 15% were aware of their right to make decisions regarding seeking maternal health services and fewer than one-tenth of the women mentioned the right to information as one of their rights related to maternal health.

**Table 2:** Awareness and perceptions of women with a recent history of birth regarding human rights related to maternal health (N = 1367)

	Percent
**Awareness regarding rights and duty-bearers:**	
Aware of rights related to maternal health in general	71.0
Aware of specific rights related to maternal health-	
Access to quality maternal health services	40.1
Respectful maternity care from health service providers	20.5
Decide to seek health services autonomously	13.2
Information	9.3
Family planning	11.0
Freedom from violence	11.1
Aware of government's role as duty-bearer	20.4
Aware of husbands’ role as duty-bearers	51.4
Aware of family members' role as duty-bearers	32.0
Aware of communities' role as duty-bearers	10.8
**Perception regarding the realization of rights:**	
Perceive that women in the community are able to realize their rights	10.1
Perceive that the woman herself is able to realize her rights	29.5
Perceive that the government fulfils its role as duty-bearer	27.1
Perceive that husbands fulfil their role as duty-bearers	36.9
Perceive that families fulfil their role as duty-bearers	23.0
Perceive that communities fulfil their role as duty-bearers	9.6

**Figure 1:**
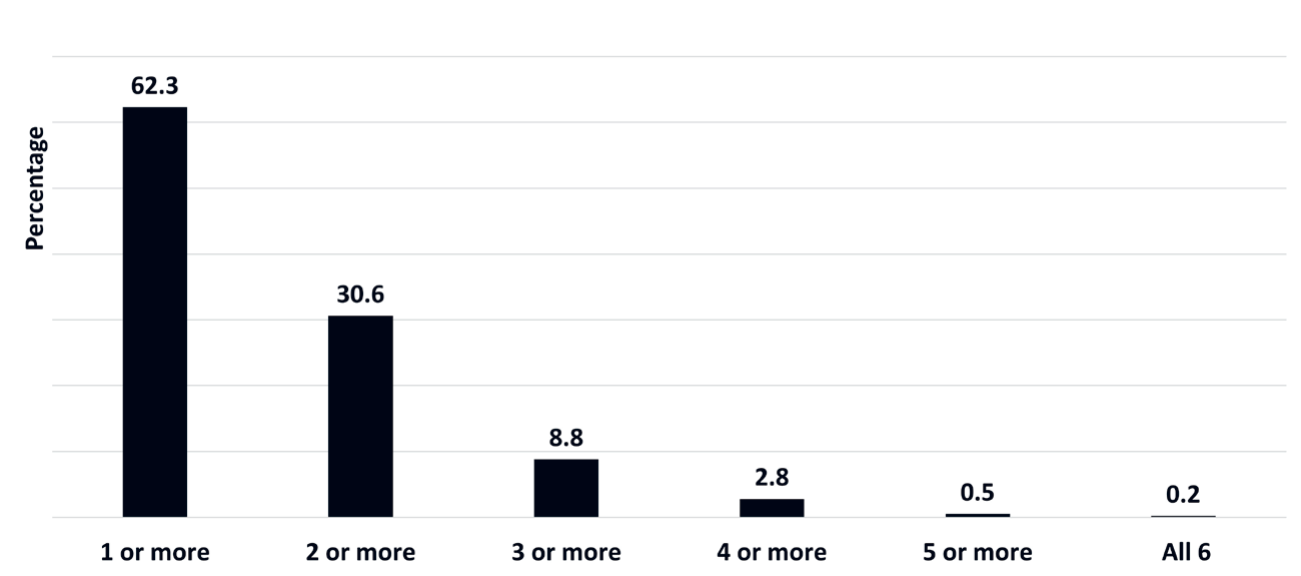
Awareness regarding human rights related to maternal heath among women with a recent history of birth (N = 1367).

Just under one-third of the women mentioned two or more specific human rights related to maternal health and less than 10% mentioned three or more of these specific human rights (**Figure 1**).

Only one in every five women mentioned that the government has a role as a duty-bearer for respecting, protecting and fulfilling human rights related to maternal health. Respondents recognized moral duty-bearers more readily. Half of the respondents mentioned husbands as duty-bearers and one-third of the women mentioned families as duty-bearers in this regard. Approximately one-tenth of the women perceived that women in their communities could realize their rights related to maternal health. However, around one-third reported that they individually could realize their rights related to maternal health. Around 27% of women reported that the government fulfils its role as duty-bearers. Perception regarding the husbands fulfilling the roles of duty-bearers was slightly higher (37%).

**Table 3** presents the relationship between background characteristics and awareness and perceptions regarding human rights related to maternal health. Higher educational attainment (10 or more years of formal education) was significantly associated with awareness regarding human rights related to maternal health in general (AOR = 1.7; 95% CI = 1.1-2.7). Higher educational attainment was also significantly associated with awareness of three or more specific human rights related to maternal health (AOR = 2.0; 95% CI = 1.1-5.0). Similarly, higher education was associated with awareness regarding the role the government (AOR = 2.0; 95% CI = 1.2-3.2) and husbands (AOR = 1.6; 95% CI = 1.1-2.3) as duty-bearers with obligations and responsibilities for respecting, protecting and fulfilling human rights related to maternal health. Religion (being non-Muslim) was significantly associated with awareness of the role of the government as a duty-bearer in fulfilling rights (AOR = 2.9; 95% CI = 1.5-5.8). Although religion (being non-Muslim) was associated with awareness of three or more specific human rights in binary logistic regression models (OR = 2.6; 95% CI = 1.1-6.1), significance was lost after adjusting for background characteristics (AOR = 2.2; 95% CI = 0.9-5.3). Belonging to the highest two wealth quintiles was significantly associated with awareness regarding three or more specific human rights related to maternal health (Fourth wealth quintile: AOR = 2.2; 95% CI = 1.0-4.9; Highest wealth quintile: 3.5; 95% CI = 1.6-7.6). Similarly, wealthier women were more likely to perceive that they were able to realize their rights related to maternal health (Fourth wealth quintile: AOR = 2.1; 95% CI = 1.4-3.2; Highest wealth quintile: AOR = 2.5; 95% CI = 1.6-3.9). Wealthier women were also more likely to report that their husbands fulfilled their roles as duty-bearers in contributing to their realization of human rights (Fourth wealth quintile: AOR = 1.6; 95% CI = 1.1-2.3; Highest wealth quintile: 1.7; 95% CI = 1.2-2.6), as well as that the government fulfilling its role as a duty-bearer (Fourth wealth quintile: AOR = 2.1; 95% CI = 1.4-3.2; Highest wealth quintile: 1.9; 95% CI = 1.2-2.9).

**Table 3:** Relationship between background characteristics and awareness regarding rights and perceptions regarding the realization of rights

Background characteristic	Aware of rights related to maternal health in general			Aware of 3 or more specific rights			Aware of government's role as duty-bearers			Aware of husbands’ role as duty-bearers			Perceive that the woman herself is able to realize her rights			Perceive that the government fulfils its role as duty-bearer			Perceive that husbands fulfil their role as duty-bearers		
	**%**	**OR (CI)**	**AOR (CI)**	**%**	**OR (CI)**	**AOR (CI)**	**%**	**OR (CI)**	**AOR (CI)**	**%**	**OR (CI)**	**AOR (CI)**	**%**	**OR (CI)**	**AOR (CI)**	**%**	**OR (CI)**	**AOR (CI)**	**%**	**OR (CI)**	**AOR (CI)**
**Woman’s age (years):**																					
15-24	72.2	**ref**	**ref**	9.1	**ref**	**ref**	18.9	**ref**	**ref**	53.2	**ref**	**ref**	34.1	**ref**	**ref**	26.6	**ref**	**ref**	39.6	**ref**	**ref**
25-34	71.0	0.9 (0.7,1.2)	1.2 (0.9,1.5)	9.0	1.0 (0.7,1.5)	1.4 (0.9,2.2)	22.4	1.2 (0.9,1.6)	1.3 (0.9,1.8)	50.9	0.9 (0.7,1.1)	1.2 (0.9,1.5)	26.1	0.7 (0.5,0.9)	0.7 (0.5,0.9)	27.6	1.1 (0.8,1.4)	1 (0.7,1.4)	35.2	0.8 (0.7,1.0)	1.0 (0.7,1.2)
35≥	63.0	0.7 (0.4,1.0)	0.8 (0.5,1.4)	5.6	0.6 (0.2,1.4)	1.0 (0.4,2.7)	18.5	1.0 (0.6,1.6)	1.0 (0.6,1.9)	42.6	0.7 (0.4,1)	0.9 (0.6,1.5)	19.4	0.5 (0.3,0.8)	0.5 (0.3,0.9)	26.9	1 (0.6,1.6)	0.9 (0.6,1.6)	30.6	0.7 (0.4,1)	0.8 (0.5,1.3)
**Woman’s education (years):**																					
0-4	66.4	**ref**	**ref**	3.5	**ref**	**ref**	18.4	**ref**	**ref**	44.2	**ref**	**ref**	18.0	**ref**	**ref**	23.0	**ref**	**ref**	31.4	**ref**	**ref**
5-9	69.0	1.1 (0.8,1.5)	1.0 (0.7,1.4)	9.0	2.7 (1.4,5.3)	2.0 (1.0-4.0)	18.3	1.0 (0.7,1.4)	1.0 (0.7,1.5)	50.8	1.3 (1.0,1.7)	1.2 (0.8,1.6)	28.4	1.8 (1.3,2.5)	1.3 (0.9,2.0)	27.9	1.3 (0.9,1.8)	1.2 (0.8,1.6)	37.0	1.3 (1.0,1.7)	1.1 (0.8,1.5)
≥10	80.8	2.1 (1.4,3.1)	1.7 (1.1,2.7)	13.3	4.2 (2,8.6)	2.0 (1.1,5.0)	28.3	1.8 (1.2,2.6)	2.0 (1.2,3.2)	60.1	1.9 (1.4,2.7)	1.6 (1.1,2.3)	43.7	3.5 (2.4,5.2)	2.0 (1.3,3.2)	28.7	1.3 (0.9,2.0)	1.0 (0.6,1.6)	42.3	1.6 (1.1,2.3)	1.1 (0.7,1.7)
**Religion:**																					
Muslim	70.8	**ref**	**ref**	8.5	**ref**	**ref**	19.8	**ref**	**ref**	51.7	**ref**	**ref**	29.5	**ref**	**ref**	27.2	**ref**	**ref**	37.3	**ref**	**ref**
Other	75.0	1.2 (0.6,2.6)	1.1 (0.5,2.3)	19.4	2.6 (1.1,6.1)	2.2 (0.9,5.3)	41.7	2.9 (1.5,5.7)	2.9 (1.5,5.8)	38.9	0.6 (0.3,1.2)	0.5 (0.2,1)	30.6	1.1 (0.5,2.2)	0.9 (0.4,1.9)	22.2	0.8 (0.3,1.7)	0.7 (0.3,1.6)	25.0	0.6 (0.3,1.2)	0.5 (0.2,1.1)
**Parity:**																					
1	76.2	**ref**	**ref**	11.2	**ref**	**ref**	19.9	**ref**	**ref**	58.5	**ref**	**ref**	32.3	**ref**	**ref**	26.7	**ref**	**ref**	43.4	**ref**	**ref**
2	70.8	0.8 (0.6,1)	0.7 (0.5,1)	9.5	0.8 (0.5,1.3)	0.8 (0.5,1.3)	19.4	1.0 (0.7,1.4)	1.0 (0.7,1.4)	51.7	0.8 (0.6,1)	0.7 (0.6,1.0)	32.1	1.0 (0.7,1.3)	1.1 (0.8,1.6)	27.6	1.0 (0.8,1.4)	1.1 (0.8,1.5)	35.5	0.7 (0.5,0.9)	0.7 (0.5,0.9)
3	66.3	0.6 (0.4,0.9)	0.6 (0.4,0.9)	6.8	0.6 (0.3,1.0)	0.6 (0.3,1.1)	22.0	1.1 (0.8,1.7)	1.1 (0.7,1.7)	44.7	0.6 (0.4,0.8)	0.6 (0.4,0.8)	26.9	0.8 (0.5,1.1)	1.1 (0.7,1.6)	24.2	0.9 (0.6,1.3)	0.9 (0.6,1.4)	30.3	0.6 (0.4,0.8)	0.6 (0.4,0.9)
4≥	67.8	0.7 (0.5,0.9)	0.7 (0.5,1.1)	6.4	0.5 (0.3,0.9)	0.7 (0.3,1.3)	21.2	1.1 (0.8,1.6)	1.1 (0.7,1.8)	46.6	0.6 (0.5,0.8)	0.6 (0.4,0.9)	24.4	0.7 (0.5,0.9)	1.3 (0.8,2.0)	29.6	1.2 (0.8,1.6)	1.3 (0.9,2.0)	35.0	0.7 (0.5,1.0)	0.8 (0.6,1.2)
**Wealth quintile:**																					
Lowest	67.2	**ref**	**ref**	3.6	**ref**	**ref**	17.9	**ref**	**ref**	46.7	**ref**	**ref**	18.2	**ref**	**ref**	20.4	**ref**	**ref**	29.9	**ref**	**ref**
Second	68.5	1.1 (0.7,1.5)	1.0 (0.7,1.5)	6.2	1.8 (0.8,3.9)	1.5 (0.7,3.4)	20.5	1.2 (0.8,1.8)	1.2 (0.8,1.9)	47.6	1.0 (0.7,1.5)	1 (0.7,1.4)	22.7	1.3 (0.9,2.0)	1.2 (0.8,1.8)	23.1	1.2 (0.8,1.8)	1.2 (0.8,1.8)	34.4	1.2 (0.9,1.8)	1.2 (0.8,1.8)
Middle	69.7	1.1 (0.8,1.6)	1 (0.7,1.5)	8.0	2.3 (1.1,5)	1.8 (0.8,3.9)	21.9	1.3 (0.8,2)	1.2 (0.8,1.9)	50.4	1.2 (0.8,1.6)	1.0 (0.7,1.5)	26.6	1.6 (1.1,2.4)	1.4 (0.9,2.1)	25.9	1.4 (0.9,2.0)	1.3 (0.9,2.0)	34.3	1.2 (0.9,1.8)	1.2 (0.8,1.7)
Fourth	72.5	1.3 (0.9,1.9)	1.1 (0.7,1.6)	10.3	3.0 (1.4,6.3)	2.2 (1.1,4.9)	19.4	1.1 (0.7,1.7)	0.9 (0.6,1.5)	54.9	1.4 (1,1.9)	1.2 (0.8,1.7)	36.6	2.6 (1.7,3.8)	2.1 (1.4,3.2)	34.4	2.0 (1.4,3)	2.1 (1.4,3.2)	41.8	1.7 (1.2,2.4)	1.6 (1.1,2.3)
Highest	76.9	1.6 (1.1,2.4)	1.3 (0.8,1.9)	15.8	4.9 (2.4,10.0)	3.5 (1.6,7.6)	22.3	1.3 (0.9,2.0)	1.0 (0.6,1.6)	57.1	1.5 (1.1,2.1)	1.2 (0.8,1.7)	43.2	3.4 (2.3,5.0)	2.5 (1.6,3.9)	31.5	1.8 (1.2,2.6)	1.9 (1.2,2.9)	44.3	1.9 (1.3,2.7)	1.7 (1.2,2.6)

OR – odds ratio, AOR – adjusted odds ratio, CI – confidence interval

**Table 4** presents the relationship between awareness of human rights related to maternal health and perceptions around the realization of human rights and utilization of maternal health services. Awareness of rights related to maternal health in general was significantly associated with attending four or more ANC contacts (with formal health care providers) (AOR = 1.7, 95% CI = 1.1-2.8). Awareness of three or more specific human rights was also positively associated with attending four or more ANC contacts (AOR = 2.4; 95% CI = 1.4 -4.0). The likelihood of attending four or more ANC contacts was higher when the woman perceived that she could realize her rights (AOR = 2.1; 95% CI = 1.4-3.2). Although awareness of three or more specific human rights related to maternal health (OR = 1.5; 95% CI = 1.1-2.2) and the government’s role as duty-bearer (OR = 1.3; 95% CI = 1.0-1.7; *P* = 0.06) were associated with giving birth in presence of an SBA in binary logistic regression models, the associations were not significant after adjusting for confounders and covariates. Similar trends were observed for the relationship between perceptions regarding the individual-level realisation of human rights and giving birth in presence of a skilled attendant (OR = 1.5, 95% CI = 1.2-2.0)

**Table 4:** Relationship between awareness and perceptions of rights related to maternal health and utilization on maternal services (N = 1367)

	Birth in presence of an SBA				4 or more ANC from formal HCP			
	**%**	**UOR**	**AOR**		**%**	**UOR**	**AOR**
		**(95% CI)**	**(95% CI)**			**(95% CI)**	**(95% CI)**
Aware of rights related to maternal health in general	(Ref) No	37.5	1.2 (0.9-1.5)	1.0 (0.7-1.3)	(Ref) No	5.3	2.0 (1.2-3.2)	1.7 (1.1-2.8)
	Yes	42.0			Yes	9.9		
Aware of 3 or more specific rights related to maternal health	(Ref) No	39.8	1.5 (1.1-2.2)	1.0 (0.7-1.6)	(Ref) No	7.4	3.3 (2.0-5.4)	2.4 (1.0-4,4)
	Yes	50.0			Yes	20.8		
Aware of Government's role as duty-bearers	(Ref) No	39.4	1.3 (1.0-1.7)	1.2 (0.9-1.5)	(Ref) No	8.1	1.3 (0.8-2.1)	1.2 (0.8-1.9)
	Yes	45.5			Yes	10.4		
Aware of husbands' role as duty-bearers	(Ref) No	40.2	1.0 (0.8-1.3)	0.8 (0.7-1.1)	(Ref) No	6.8	1.6 (1.1-2.3)	1.4 (0.9-2.1)
	Yes	41.2			Yes	10.3		
Perceive that the woman herself is able to realize her rights	(Ref) No	37.6	1.5 (1.2-2.0)	1.1 (0.9-1.5)	(Ref) No	5.8	2.9 (2.0-4.2)	2.1 (1.4-3.2)
	Yes	48.1			Yes	15.1		

SBA – skills birth attendant, UOR – unadjusted odds ratio, AOR – adjusted odds ratio, CI – confidence interval, HCP – health care provider

## DISCUSSION

Mirroring the trends in international development cooperation, maternal health is increasingly being framed as a human rights issue and human rights-based approaches to improving maternal health are gaining momentum globally and at national levels [3,7,8]. In Bangladesh, a human rights-based approach has been integrated within the maternal health strategy, where promoting women’s rights and improving equity is set as one of the guiding principles [14]. One essential component of a human rights-based approach is building the awareness of the rights-holders of their rights, which is important to developing their capacity to claim their rights [8,11]. Indeed, WHO recommends promotion of awareness of human rights at the community level as one of the health promotion interventions for improving maternal and newborn health [11]. However, little is understood regarding the awareness of human rights and perception about the realization of these rights in settings with limited resources [11,20,21]. This study addresses this key evidence gap in the context of rural Bangladesh, suggesting that while there is a reasonably high level of general awareness of human rights related to maternal health, the understanding of the specificities of these rights is somewhat limited. Moreover, perceptions regarding women’s realization of these rights and duty-bearers’ fulfilment of their obligations to respect, protect and fulfil these rights are poor. These results are especially important, as we found awareness and perceptions regarding these rights to be significantly associated with the use of skilled care during pregnancy, considered the cornerstone service for improving health outcomes of both women and newborns.

Promoting the awareness of human rights is an important step in building capacities of women to demand and claim their rights. The majority of respondents in our study were aware that women have rights related to maternal health. This suggests that the concepts of human rights related to maternal health are appropriate for initiating dialogue with women in rural contexts. However, few participants were able to mention specific human rights which have been identified in global policies and human rights instruments. Few studies have documented awareness of human rights related to maternal health, but our results are similar to what has been found in another area of rural Bangladesh [22]. Our results are also consistent with studies carried out in China, exploring awareness of gender concepts and gender equity, closely related to human rights, which found a generally low level of awareness related to gender equity [23-25]. Building awareness of human rights and gender equity in resource-constrained settings like Bangladesh should be considered a priority for moving toward gender equality and realization of human rights as envisioned in global and national maternal health strategies [14].

Within a human rights-based framework, it is critical for rights-holders to not only be aware of their rights but also of the obligations and responsibilities of duty-bearers to respect, protect and fulfil these rights. This awareness is requisite for rights-holders to know where they can turn to claim their rights and to be equipped to hold duty-bearers accountable. Government is the primary duty-bearer, with binding obligations to respect, protect and fulfil the rights of women related to maternal health [3,7]; therefore the governments should be taking appropriate measures to avert preventable maternal mortality and morbidity. High maternal mortality and morbidity in countries reflect a country’s failure to prioritize the human rights and needs of women. Interestingly, only one-fifth of participants mentioned spontaneously the government as a duty-bearer. However, more respondents mentioned husbands and family members as duty-bearers which may indicate a primary reliance on families and social networks over the government for meeting maternal health needs and demands. This explanation can be supported by the fact that village doctors and informal health care providers are the most dominant health care providers in rural Bangladesh, despite community awareness of their limitations with regard to knowledge and skills [26,27]. The explanation is further supported by the very high use of traditional birth attendants in rural Bangladesh [13,17]. These results also highlight that women and communities need to be better informed regarding the obligations and responsibilities of the government as the primary duty-bearer in fulfilment of rights related to maternal health. For instance, they should know that the Ministry of Health has the obligation to provide quality maternal health services and that this is not an act of charity, and that government should be held accountable for providing these services.

Appropriate strategies should be explored for the promotion of this awareness in communities. Not surprisingly, we found a significant association between the awareness of human rights related to maternal health and educational attainment and wealth status, which indicates the relative lack of awareness in communities with less access to education and wealth. This emphasizes the need for targeted actions in order to build awareness of rights in the most marginalized of societies and create an enabling environment for them to realize their rights, as they are less likely to be able to do so. The high coverage of at least one ANC contact from a formal health care provider, and with relatively fewer equity gaps in such coverage, present ANC as a promising platform for promotion of human rights related to maternal health. In Bangladesh, ANC is also provided through a large network of domiciliary health workers, which may act as a key strategy to reach the marginalized population for the promotion of human rights awareness. Although the coverage of post-natal care is sub-optimum and wide equity gaps in the coverage of this service persist, it may still be an important platform for the promotion of human rights through the routine health systems. Further research is required to develop and test innovative approaches to effectively promote awareness of human rights in marginalized populations as this is particularly a weak area in the evidence related sexual and reproductive health and rights [28].

With regard to perceptions around the realization of human rights, less than one-third of women in our study responded that they feel that they are able to satisfactorily realize their rights related to maternal health. Interestingly, only 10% indicated that they felt that other women in the broader community are able to realize their rights related to maternal health. This may indicate that even when women themselves feel that they are in a position to realize their own rights, they are cognizant of the plight of other women around them and the challenges that they face, particularly in marginalized and socially excluded groups. In terms of the perceptions of the roles of duty-bearers, only a quarter of our study population expressed that the government satisfactorily fulfils its obligations as a duty-bearer, once the concept had been explained to them during the survey. Social accountability initiatives may be one avenue for increasing the awareness of communities regarding the roles of the government as the primary duty-bearers, and for allowing communities to be involved in holding the government accountable for fulfilling these obligations [29]. Such community engagement interventions are increasingly being experimented within a variety of settings to hold the governments accountable in the provision of sexual and reproductive health services, including maternal health services. These experiences have demonstrated both potential and challenges [29-34]. In the context of Bangladesh, each community clinic (primary health care centre for around every 6000 population) is staffed by a minimally-trained government employed health worker and managed by a committee which is constituted by representatives from the surrounding communities. These committees could serve as a platform to promote accountability within health systems at the local level with regard to provision of maternal health services. It is also essential to work with health service managers and providers, building their awareness of their roles and responsibilities as duty-bearers at the interface with women and communities and their capacities to meet these obligations. Initiatives to promote respectful maternity care and baby-friendly health services could also contribute in this regard. These initiatives are already included in the maternal health strategy of Bangladesh, however, implementation needs to be reinforced [14].

It is also important to recognize the valuable contributions which can be made by moral duty-bearers and the importance of building their capacities to be able to do so [35]. While half of the respondents mentioned husbands as duty-bearers for contributing to the respect, protection and fulfilment of human rights related to maternal health, just over one-third of the women reported that husbands satisfactorily fulfil their roles. This is of critical importance in patriarchal societies, such as that in rural Bangladesh, as in many cases men play a deciding role in determining whether or not women are able to access maternal health services [35,36]. A rights-based approach to maternal health programming should also take this into consideration and aim to build the capacities of men as moral duty-bearers to contribute to assisting women in the realization of these rights. However, interventions aiming to influence the role of men in maternal health should be carefully considered and assessed in a gender transformative way, which do not compromise the autonomy and decision-making power of women in such contexts [35,37-39]. Such unintended consequences could undermine the objectives of a human rights-based approach. It is to be noted that the findings presented in this paper regarding the role of the duty-bearers are the subjective interpretations of women. While there are other measures for assessing the degree to which duty-bearers fulfil their responsibilities and obligations, it is critical to consider the subjective perspectives and opinions of women in this regard, as it is their rights which are at stake. In addition, this knowledge is imperative to build the capacity of rights holders to claim their rights from duty-bearers.

Promisingly, our findings suggest that awareness and perceptions of human rights related to maternal health are associated with the use of skilled maternal health services. This association was particularly strong in relation to care during pregnancy, where significant associations were found in all categories of awareness and perception with women attending at least four ANC contacts. We also found a significant association between awareness and perceptions regarding human rights related to maternal health and birth with a skilled attendant in binary logistic regression models; however, they were not significant after adjustment. It is possible that we did not find an increase in birth with an SBA given the strong preference for home birth in the Bangladesh context [40]. Indeed, even when women are aware of their rights, they may still decide to give birth in the home setting, particularly if they feel that the care which they receive in this setting is more respectful and better responds to their preferences. Studies exploring human rights related to maternal health have primarily been conducted in intervention settings, ie, measuring how the promotion of rights effects the use of skilled health services following an intervention. These studies have generally found an association between the promotion of human rights and increased use of ANC [22,30,41,42]. Our study adds to this body of knowledge by demonstrating similar associations. Our findings are also consistent with the studies conducted in China, which have found that awareness of human rights and gender issues among women is associated with increased utilization of skilled care during pregnancy and at the time of birth [23-25]. Taken together, these studies along with our findings can serve as a guide to programmers in developing appropriate interventions for promotion of awareness of rights for improving maternal health. However, promotion of the awareness of human rights should not be implemented as a stand-alone intervention. Rather, it should be nested within a broader human rights-based approach, moving toward equitable social structures which address the needs of the marginalized, promote enabling policy environments, strive for better provision and quality of health services, and ensure accountability [8,10]. Considering a human rights-based approach to maternal health as a larger development issue, the health systems will also require building strong and effective intersectorial collaboration between different agencies of the government and other stakeholders to respect, protect and fulfil these rightcs [8,43,44].

Further research is warranted at the local level to understand perceptions of rights related to maternal health as a human rights-based framing is recommended globally and nationally. Moreover, as human rights-based initiatives to maternal and newborn health increasingly take shape, it is of critical importance to carefully document these and generate evidence linking inputs and actions to health and human-rights outcomes.

### Limitations

It is important to acknowledge some of the limitations of our study and outline the strategies that we adopted to address them. First, the results presented in this paper are from a cross-sectional survey and we cannot infer causality for the associations that we have presented. We have tried to adjust for the possible effect of the confounders by presenting AORs with multiple regression models. We also acknowledge the potential of recall bias in our study. Based on the pregnancy outcomes, the women’s perception regarding the roles of duty-bearers may have changed. We asked the questions related to rights before asking the questions related to the utilization of maternal health services to minimize this bias. Recall error is also another potential limitation of this study, as we accepted up to 12 months of recall. However, we feel that the data collectors were well trained and had the capacity to clarify different elements of the questionnaire to respondents for their proper understanding and appropriate recall. Moreover, the recall period in our study was 12 months, which is much shorter than the 3 to 5 years recall period that is accepted by other surveys generating national estimates [13,45,46]. Another potential limitation could be social desirability bias, which we tried to address by recruiting data collectors from local communities who are familiar with the local culture, language and norms. Lastly, the validated tools used in national surveys did not have extensive questions related to awareness and perceptions of human rights. Therefore, it was challenging to quantify the awareness and perception of human rights through a household interview survey. We acknowledge that the variables through which we have tried to operationalize rights only capture certain elements of the human rights implicated in maternal health and are not exhaustive. However, we adopted these variables based on the recommendations of the High Level Working Group for the Health and Human Rights of Women, Children and Adolescents [8]. We rigorously pre-tested the questions related to rights in rural communities to make necessary adjustments in the language and order of relevant questions, and provided standardized introductions to the questions to ensure better comprehensibility. We also acknowledge that the findings presented in our study are not representative of the total community as we only surveyed the women who had a recent history of childbirth.

## CONCLUSION

Our study findings suggest that interventions promoting the awareness of human rights related to maternal health would be appropriate within the community in our study setting. There is already a general level of awareness, which would provide a foundation, which interventions could build on. Moreover, our results suggest that such interventions are justified, as this awareness and these perceptions are directly associated with the use of skilled care during pregnancy. The Government of Bangladesh should prioritize interventions, which aim to build awareness of human rights at the community level as part of the broader application of a human rights-based approach to improving maternal health in order to move toward the realization of rights and well-being of women throughout the country.

## Additional Material

Online Supplementary Document

## References

[1] World Health Organization. UNICEF. Trends in maternal mortality: 1990-2015: estimates from WHO, UNICEF, UNFPA, World Bank Group and the United Nations Population Division. 2015.

[2] World Health Organization. Cause-specific mortality: regional estimates for 2000-2011. Geneva: WHO. 2012.

[3] United Nations Human Rights Council. Resolution of the United Nations human rights council on preventable maternal mortality and morbidity and human rights. 2009.

[4] Assembly UG. International covenant on economic, social and cultural rights. United Nations, Treaty Series. 1966; p 993.

[5] United Nations. 2030 Agenda for Sustainable Development. New York, NY: UN; 2015.

[6] United Nations Population Fund (UNFPA), editor. International conference on population and development program of action 1994. New York, NY: UNPFA; 1994.

[7] United Nations Human Rights Council. Technical guidance on the application of a human-rights based approach to the implementation of policies and programmes to reduce preventable maternal morbidity and mortality. New York, NY: UN; 2012.

[8] World Health Organization. Leading the realization of human rights to health and through health: report of the High-Level working group on the health and human rights of women, children and adolescents. 2017.

[9] United Nations. The Human Rights–Based Approach to Development Cooperation: Towards a Common Understanding among UN Agencies: Interagency Workshop on a Human Rights–Based Approach. 2003.

[10] Peters DH Garg A Bloom G Walker DG Brieger WR Rahman MH Poverty and access to health care in developing countries. Ann N Y Acad Sci. 2008;1136:161-71. 10.1196/annals.1425.011 17954679

[11] World Health Organization. WHO recommendations on health promotion interventions for maternal and newborn health 2015. Geneva: WHO; 2015. 26180864

[12] El Arifeen S Hill K Ahsan KZ Jamil K Nahar Q Streatfield PK Maternal mortality in Bangladesh: a Countdown to 2015 country case study. Lancet. 2014;384:1366-74. 10.1016/S0140-6736(14)60955-7 24990814

[13] National Institute of Population Research and Training (NIPORT), International Centre for Diarrhoeal Disease Research Bangladesh, MEASURE Evaluation. Bangladesh maternal mortality and health care survey (BMMS) 2016: Preliminary Report. Dhaka, Bangladesh and Chapel Hill, NC, USA: 2017.

[14] Government of Bangladesh. Maternal Health Strategy of Bangladesh. Ministry of Health and Family Welfare. 2017.

[15] National Institute of Population Research and Training (NIPORT), Mitra and Associates, ICF International. Bangladesh Demographic and Health Survey 2011. Dhaka, Bangladesh and Calverton, Maryland, USA: 2013.

[16] National Institute of Population Research and Training, MEASURE Evaluation, University of North Carolina at Chapel Hill, icddr b. Bangladesh Maternal Mortalty and Health Care Survey 2010. 2012.

[17] National Institute of Population Research and Training. (NIPORT, Associates for Community and Population Research (ACPR), ICF International. Bangladesh Health Facility Survey 2014. Dhaka, Bangladesh: 2016.

[18] Vyas S Kumaranayake L Constructing socio-economic status indices: how to use principal components analysis. Health Policy Plan. 2006;21:459-68. 10.1093/heapol/czl029 17030551

[19] Wold S Esbensen K Geladi P Principal component analysis. Chemom Intell Lab Syst. 1987;2:37-52. 10.1016/0169-7439(87)80084-9

[20] George AS Branchini C Principles and processes behind promoting awareness of rights for quality maternal care services: a synthesis of stakeholder experiences and implementation factors. BMC Pregnancy Childbirth. 2017;17:264. 10.1186/s12884-017-1446-x 28854888 PMC5577669

[21] George A Chopra M Seymour D Marchi P Making rights more relevant for health professionals. Lancet. 2010;375:1764-5. 10.1016/S0140-6736(09)62102-4 20494714

[22] Rahman AEPJ Mazumder T Haider MR Siddique AB Capello C Santarelli C Capacities of women and men to improve maternal and newborn health: effect of a community-based intervention package in rural Bangladesh. J Glob Health. 2019;9:01041 10.7189/jogh.09.010415 PMC6318832 30643636

[23] Cui Y Zhang Q Yang L Ye J Lv M Effect of married women’s beliefs about gender equity on their use of prenatal and delivery care in rural China. Int J Gynaecol Obstet. 2010;111:148-51. 10.1016/j.ijgo.2010.05.021 20800839

[24] Ying C Li Y Hui H The impact of husbands’ gender equity awareness on wives’ reproductive health in rural areas of China. Obstet Gynecol Surv. 2011;66:103-8. 10.1097/OGX.0b013e31821b8de9 21592416

[25] Wang L Cui Y Zhang L Wang C Jiang Y Shi W Influence of gender equity awareness on women’s reproductive healthcare in rural areas of midwest China. Int J Gynaecol Obstet. 2013;123:155-9. 10.1016/j.ijgo.2013.05.023 23992624

[26] Ahmed SM Hossain MA RajaChowdhury AM, Bhuiya AU. The health workforce crisis in Bangladesh: shortage, inappropriate skill-mix and inequitable distribution. Hum Resour Health. 2011;9:3. 10.1186/1478-4491-9-3 21255446 PMC3037300

[27] Mahmood SS Iqbal M Hanifi S Wahed T Bhuiya A Are ‘Village Doctors’ in Bangladesh a curse or a blessing? BMC Int Health Hum Rights. 2010;10:18. 10.1186/1472-698X-10-18 20602805 PMC2910021

[28] Hartmann M Khosla R Krishnan S George A Gruskin S Amin A How are gender equality and human rights interventions included in sexual and reproductive health programmes and policies: a systematic review of existing research foci and gaps. PLoS One. 2016;11:e0167542. 10.1371/journal.pone.0167542 28002440 PMC5176262

[29] Van Belle S Boydell V George AS Brinkerhof DW Khosla R Broadening understanding of accountability ecosystems in sexual and reproductive health and rights: A systematic review. PLoS One. 2018;13:e0196788. 10.1371/journal.pone.0196788 29990361 PMC6039047

[30] Sinha D. Empowering communities to make pregnancy safer: an intervention in rural Andhra Pradesh. New York, NY: Population Council; 2008.

[31] Murthy RK Klugman B Service accountability and community participation in the context of health sector reforms in Asia: implications for sexual and reproductive health services. Health Policy Plan. 2004;19:i78-86. 10.1093/heapol/czh048 15452018

[32] Blake C Annorbah-Sarpei NA Bailey C Ismaila Y Deganus S Bosomprah S Scorecards and social accountability for improved maternal and newborn health services: a pilot in the Ashanti and Volta regions of Ghana. Int J Gynaecol Obstet. 2016;135:372-9. 10.1016/j.ijgo.2016.10.004 27784594

[33] Hulton L Matthews Z Martin-Hilber A Adanu R Ferla C Getachew A Using evidence to drive action: A “revolution in accountability” to implement quality care for better maternal and newborn health in Africa. Int J Gynaecol Obstet. 2014;127:96-101. 10.1016/j.ijgo.2014.07.002 25087502

[34] Papp SA Gogoi A Campbell C Improving maternal health through social accountability: A case study from Orissa, India. Glob Public Health. 2013;8:449-64. 10.1080/17441692.2012.748085 23230827

[35] Rahman AE Perkins J Islam S Siddique AB Moinuddin M Anwar MR Knowledge and involvement of husbands in maternal and newborn health in rural Bangladesh. BMC Pregnancy Childbirth. 2018;18:247. 10.1186/s12884-018-1882-2 29914410 PMC6007056

[36] Story WT Burgard SA Couples’ reports of household decision-making and the utilization of maternal health services in Bangladesh. Soc Sci Med. 2012;75:2403-11. 10.1016/j.socscimed.2012.09.017 23068556 PMC3523098

[37] Comrie-Thomson L Tokhi M Ampt F Portela A Chersich M Khanna R Challenging gender inequity through male involvement in maternal and newborn health: critical assessment of an emerging evidence base. Cult Health Sex. 2015;17:S177-89. 10.1080/13691058.2015.1053412 26159766 PMC4706017

[38] Char A. Male involvement in family planning and reproductive health in rural central India: Tampere University Press; 2011.

[39] Mullany BC Hindin MJ Becker S Can women’s autonomy impede male involvement in pregnancy health in Katmandu, Nepal? Soc Sci Med. 2005;61:1993-2006. 10.1016/j.socscimed.2005.04.006 15922498

[40] Sarker BK Rhman M Rahman T Hossain J Reichenback L Mitra D Reasons for preference of home delivery with traditional birth attendants (tbas) in rural Bangladesh: A qualitative exploration. PLoS One. 2016;11:e0146161. 10.1371/journal.pone.0146161 26731276 PMC4701391

[41] Pandey P Sehgal AR Riboud M Levine D Goyal M Informing resource-poor populations and the delivery of entitled health and social services in rural India: a cluster randomized controlled trial. JAMA. 2007;298:1867-75. 10.1001/jama.298.16.1867 17954538

[42] George AS Branchini C Portela A Do interventions that promote awareness of rights increase use of maternity care services? A systematic review. PLoS One. 2015;10:e0138116. 10.1371/journal.pone.0138116 26444291 PMC4596618

[43] Yamin AE Toward transformative accountability: applying rights-based approach to fulfill maternal health obligations. SUR-Int J Hum Rights. 2010;12:95.

[44] Yamin AE Applying human rights to maternal health: UN Technical Guidance on rights-based approaches. Int J Gynaecol Obstet. 2013;121:190-3. 10.1016/j.ijgo.2013.01.002 23395448

[45] MEASURE Evaluation. ICF International. Guie to DHS Statistics: Demographic and Health Survey Methodology. Calverton, Maryland 2006.

[46] National Institute of Population Research and Training (NIPORT), Mitra and Associates, ICF International. Bangladesh Demographic and Health Survey 2014. Dhaka, Bangladesh, and Rockville, Maryland, USA: 2016.

