# Supplementary material for: Awareness and perceptions of women regarding human rights related to maternal health in rural Bangladesh
Source: J Glob Health. 2019 Jun 15;9(1):010415. doi: 10.7189/jogh.09.010415 (PMC6571109; doi:10.7189/jogh.09.010415)
Supplement: Online Supplementary Document [file jogh-09-010415-s001.pdf]

J Glob Health 2019: 9: 010415

Table S1

	Sarail	Kasba	Bijoynagar
	#	#	#
Population	327311	331937	267041
Union	09	10	10
Village	141	209	225
Ave household size	5.36	5.23	5.28
Upazila Health Complex (Hospital)	9	2	10
Upazila Health and Family Welfare Centre	4	7	10
Community Clinic	21	32	28
Health Assistant (Community Health Worker for health promotion and vaccination services)	44	32	25
Family Welfare Assistant (Community Health Worker for Family Planning services)	50	50	31
Community Health Care Provider (Community Health Worker for Community Clinics)	23	32	28
